# Adhesion and Cohesion of Silica Surfaces with Quartz Cement: A Molecular Simulations Study

**DOI:** 10.1021/acsomega.2c01129

**Published:** 2022-06-23

**Authors:** Sameer Al-Hajri, Daniel Bahamon, Md Motiur Rahman, Mohammed Haroun, Lourdes F. Vega

**Affiliations:** † Petroleum Engineering Department, Khalifa University, Abu Dhabi 127788, United Arab Emirates; ‡ Chemical Engineering Department, Khalifa University, Abu Dhabi 127788, United Arab Emirates; § Research and Innovation Center on CO_2_ and Hydrogen (RICH Center), Khalifa University, Abu Dhabi 127788, United Arab Emirates

## Abstract

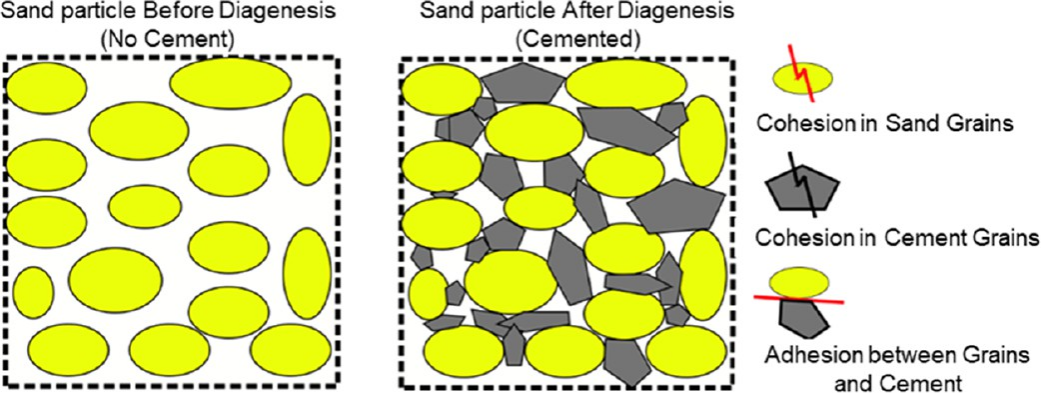

This study focuses on developing an adhesive and cohesive molecular modeling approach to study the properties of silica surfaces and quartz cement interfaces. Atomic models were created based on reported silica surface configurations and quartz cement. For the first time, molecular dynamics (MD) simulations were conducted to investigate the cohesion and adhesion properties by predicting the interaction energy and the adhesion pressure at the cement and silica surface interface. Results show that the adhesion pressure depends on the area density (per nm^2^) and degree of ionization, and van der Waals forces are the major contributor to the interactions between the cement and silica surfaces. Moreover, it is shown that adhesion pressure could be the actual rock failure mechanism in contrast to the reported literature which considers cohesion as the failure mechanism. The bonding energy factors for both “dry” and “wet” conditions were used to predict the water effect on the adhesion pressure at the cement–surface interface, revealing that H_2_O can cause a significant reduction in adhesion pressure. In addition, relating the adhesion pressure to the dimensionless area ratio of the cement to silica surfaces resulted in a good correlation that could be used to distribute the adhesion pressure in a rock system based on the area of interactions between the cement and the surface. This study shows that MD simulations can be used to understand the chemomechanics relationship fundamental of cement–surfaces of a reservoir rock at a molecular/atomic level and to predict the rock mechanical failure for sandstones, limestones, and shales.

## 1 Introduction

The mechanical failure of different formation rocks such as sandstones, limestones, and shales has been extensively investigated in the literature.^1−3^ Although the mechanical failure of formation can be successfully estimated, a set of information is required for the prediction of geomechanical properties to understand the behavior of a reservoir rock system. Poisson’s ratio, shear modulus, Young’s modulus, stress intensity factor, and all related formation stress parameters are required to be evaluated to predict the behavior and failure of a reservoir rock.^4−8^ This can be a long and costly experimental process requiring complex equipment. Moreover, conventional techniques such as experimental procedures and well logging give limited information about quartz cementation and interactions with surfaces.

For improvement and customization of cementing material properties, it is crucial to understand the nano- or microstructure of the material. This is simply a result of the fact that bulk systems are formed by smaller-scale atoms and molecules; thus, the atomic scale represents the properties of the overall system.^9^ Molecular dynamics (MD) simulation is an emerging tool that computes the physical motion of molecules and atoms with consideration of the potential energy and a known location of these molecules and atoms yielding atomic forces using Newton’s classical mechanics.^10^ MD is able to determine statistical parameters of bulk systems as it is able to simulate larger systems with extended simulation times depending on the accuracy required.^11^ The importance and advantages of molecular dynamic simulations are to provide a fundamental observation of initiation and evolution of the damage of materials at the nanoscale which is not possible to comprehend using the experimental procedures, as well as to further explore conditions difficult to evaluate experimentally. In fact, MD simulations have been recently used in modeling composites of cement to investigate the mechanical properties of various materials.^12−14^ The literature suggests that it is of great importance to study the interface interaction mechanisms of materials to rationally evaluate the stripping and adhesion of different materials.^15^ For instance, Xu and co-workers^16^ reported a thermodynamic model based on molecular simulations for investigating the adhesion and cohesion energies of concrete asphaltene, with the results showing a good agreement with the reported experimental values. In addition, snapshots of the simulations showed that MD is a useful tool for designing such materials and predicting their performance. All of these results can be considered a good indication of the effectiveness of MD in predicting the mechanical behavior of sandstones and other types of formations.^17,18^

The sandstone system is reported as an assembly of bonded grains/particles with bonding strength controlled by the cementing material between the grains.^19^ The sand pore space is filled by cementing material such as quartz that holds the grains together and reduces the voidage volume in the porous media.^20−22^ Quartz cement is the most abundant cementing material in sandstones.^23^ At a temperature of more than 75 °C, quartz precipitates from the existing water in porous media, and then quartz crystal overgrowth forms continuity and connection between the loose grains in the rock system.^24,25^

Nevertheless, to the authors’ best knowledge, no reported study has been conducted on the adhesion and cohesion forces on quartz to silica surfaces. Therefore, to obtain a deeper insight into quartz cement bonding and interactions with silica surfaces, this article is devoted to giving a comprehensive understanding, at the molecular level, of the interaction behavior of cement and surfaces of reservoir rocks.

## 2 Computational Details

MD simulations are based on statistical mechanics and Newton’s laws of motion. MD forecasts the movement of each atom or molecule based on interatomic physical interactions,^26^ thus being able to calculate important features and properties of materials such as conformational change of a matter and its physical features. The properties of the materials that are studied at a molecular/atomic level can actually be scaled up to bulk materials and thus be compared with experimental studies using pre-existing equipment. Such comparison has shown excellent agreement between the results from MD simulations and experiments.^27^

In this study, MD simulations in the NVT ensemble were conducted using the Forcite module in the BIOVIA Materials Studio software.^28^ The condensed phase optimized molecular potentials for atomistic simulation studies (COMPASS) forcefield was used for calculating the atomic interactions since it has been shown in the literature that it provides reliable results for silica-based materials.^29,30^ A time-step of 0.5 fs was used in all of the simulations. A reservoir temperature of 400° K (260° F) and a reservoir pressure of 0.0275 (4000 psi) were studied.

### 2.1 Surface Preparation

Generally, silica surfaces are initially in contact with water, which will allow silica to have different functionalized groups such as Si–O^–^ (silanolate) and Si–O–Si siloxane. These groups usually transform the neutral surface to a negatively charged one for a wide range of silica surfaces. The surface obtained by water modification controls the silica behavior and chemistry,^31^ which determines major mechanisms such as adsorption, desorption, and interaction between the surface and other particles.

Silicon is usually bridged to four oxygen atoms forming a silica molecule. Nuclear magnetic resonance NMR can identify this structure as reported in the literature.^32^ The nanoscopic silica structure in this form is reported as Q^4^. Superscript 4 demonstrates the neighboring SiO_4_ group number connected to a particular silicon. Furthermore, the exposed silicon at the surface of an object is able to bond with hydroxyl groups (OH) to achieve completion of the silicon valence. This binding depends on the number of the Si–O–Si bridges, which can be of two or three groups. If the binding is made to three groups, the corresponding environment is Q^3^. On the other hand, if the binding is made with only two groups, the resulting environment is Q^2^. It is noteworthy to mention that Q^1^ for a silicon binding with only one group might occur in principle. However, this type of structure is reported to be unstable kinetically and thermodynamically as indicated in the literature.^33^ Thus, the most common surface configurations reported in the literature (Q^2^, Q^3^, and Q^4^) for silica are used in this study. It is noteworthy to mention that silicon possesses only four valence electrons; therefore, the maximum configuration is Q^4^. Thus, silica will not exist in a Q^5+^ configuration.

The silica surface models in this study were based on previously reported silica models^30,34^ in which the silanol group area density ranged from 0 to 9.4 per nm^2^ and silanol was ionized to siloxide with 0–2.35 per nm^2^. These silica models were initially created using quantum mechanics calculations with considerations of equilibrium configurations and reaction barriers. A detailed description of the methodology is available in the study reported by Emami et al.,^30^ and Table 1 summarizes each hydroxylated silica surface used in this study.

**Table 1:** Silica Models Used at Different Silanol Configurations

silica surfaces	silanol group (per nm^2^)	description
base case	0.0	rectangular quartz cell cleaved in the (0 0 1) direction.
Q^2^	9.4	created by cleavage and hydration of the (7 × 4 × 3) supercell of quartz in the (0 0 1) direction.
Q^2^-Q^3^	6.9	a surface with a mixture of Q^2^ and Q^3^ created by hydrolysis of surface silanol of the Q^3^ surface.
Q^3^	4.7	created by cleavage and hydration of the (7 × 4 × 3) supercell of α-cristobalite.
Q^3^ Amor	4.7	containing some Q^2^ and Q^4^ environments. The surface area is almost 5% larger than that of a flat surface.
Q^3^-Q^4^	2.4	a surface with a mixture of Q^3^ and Q^4^ created by partial condensation of surface silanol groups Q^3^
Q^4^	0.0	created by complete condensation of surface silanol groups and energy minimization of Q^3^.

Moreover, posterior ionization was performed in the abovementioned structures to describe each silica surface ionized with sodium as shown in Table 2. The silica surfaces were ionized with sodium ions at different concentrations. The Q^3^ surfaces were ionized with higher sodium concentrations to study the surface behavior at higher degrees of ionization.

**Table 2:** Silica Models Used at Different Ionized Silanol Configurations

silica surface	silanol (per nm^2^)	SiO^–^ Na^+^ (per nm^2^)	description
Q^2^-1	9.4	0.84	9% ionization
Q^2^-2		1.69	18% ionization
Q^2^-Q^3^-1	6.9	0.60	9% ionization
Q^2^-Q^3^-2		1.29	18% ionization
Q^3^-1	4.7	0.25	5% ionization
Q^3^-2		0.42	9% ionization
Q^3^-3		0.67	14% ionization
Q^3^-4		0.84	18% ionization
Q^3^-5		1.26	27% ionization
Q^3^-6		2.35	50% ionization
Q^3^ Amor-1	4.7	0.45	9% ionization
Q^3^ Amor-2		0.85	18% ionization
Q^3^-Q^4^-1	2.4	0.34	15% ionization
Q^3^-Q^4^-2		0.68	30% ionization

### 2.2 Cohesion Model

**Figure 1:**
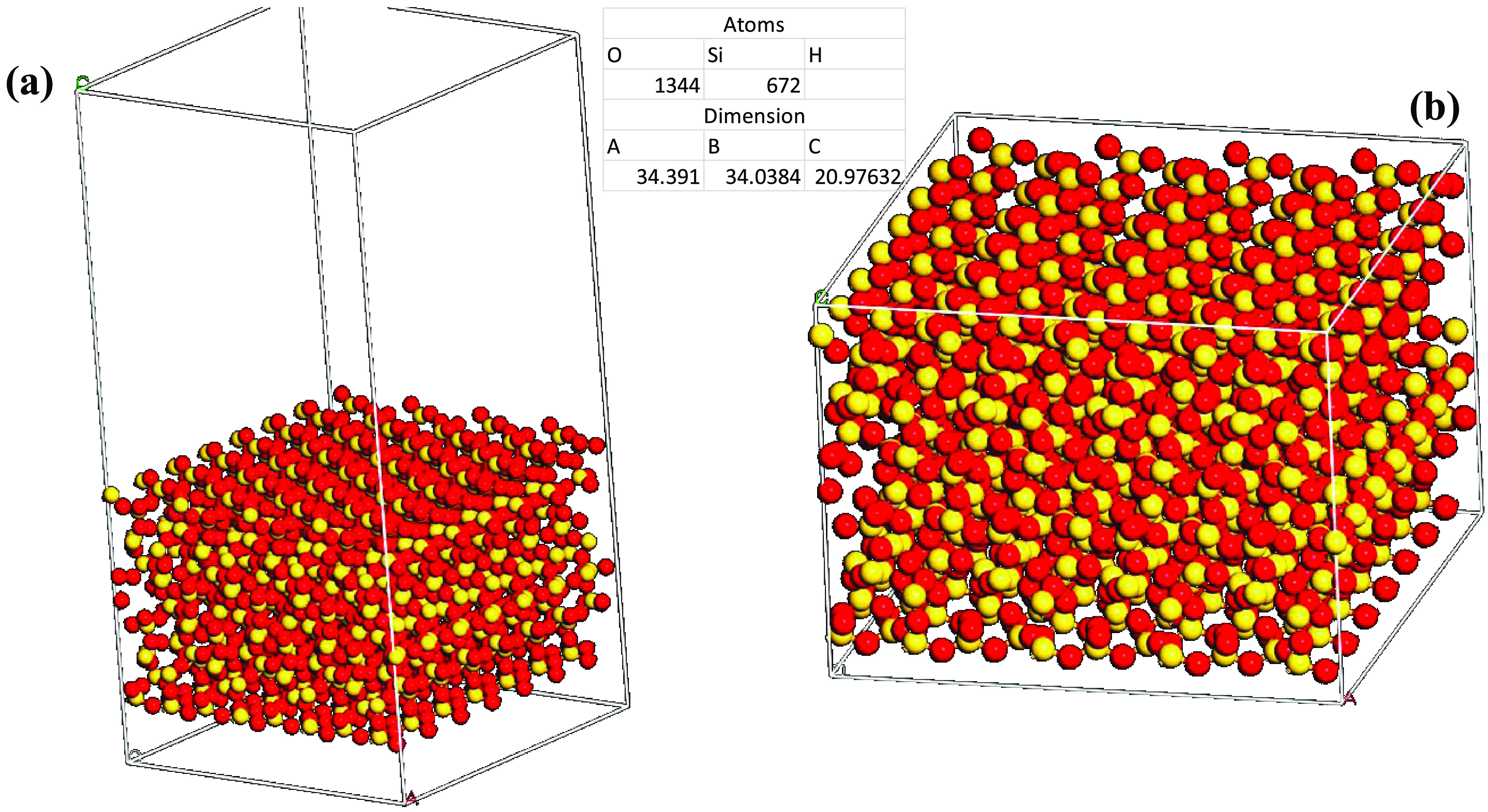
Illustration of the silica and cement cohesion models for the (a) film and (b) bulk models. Silicon (672 atoms), yellow and oxygen (1344 atoms), red.

The work of cohesion energies has been previously reported in the literature using MD simulations,^16,35^ with values showing good agreement with experimental work. Therefore, in this study, a film (confined) and a bulk model for the silica surface and cement were created, respectively, as shown in Figure 1. Both models were similar in structure but with a difference in the periodicity in the *z* direction, allowing for the top layer of the film model to act as a repulsive boundary.

Initially, each model was optimized, followed by a constant NVT simulation at reservoir pressure and temperature. To reach equilibrium, at least 200 ps were needed for each system. The surface free energy of the film and bulk models was then calculated using eq 1(16)
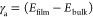
1where γ_a_ is the surface free energy, and *E*_film_ and *E*_bulk_ are the potential energies of the film and bulk cement/surface, respectively. The pressure of cohesion was then calculated using eq 2
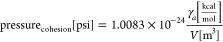
2where pressure_cohesion_ is the cohesion pressure at which mechanical failure of the surface or cement occurs.

### 2.3 Adhesion Model

**Figure 2:**
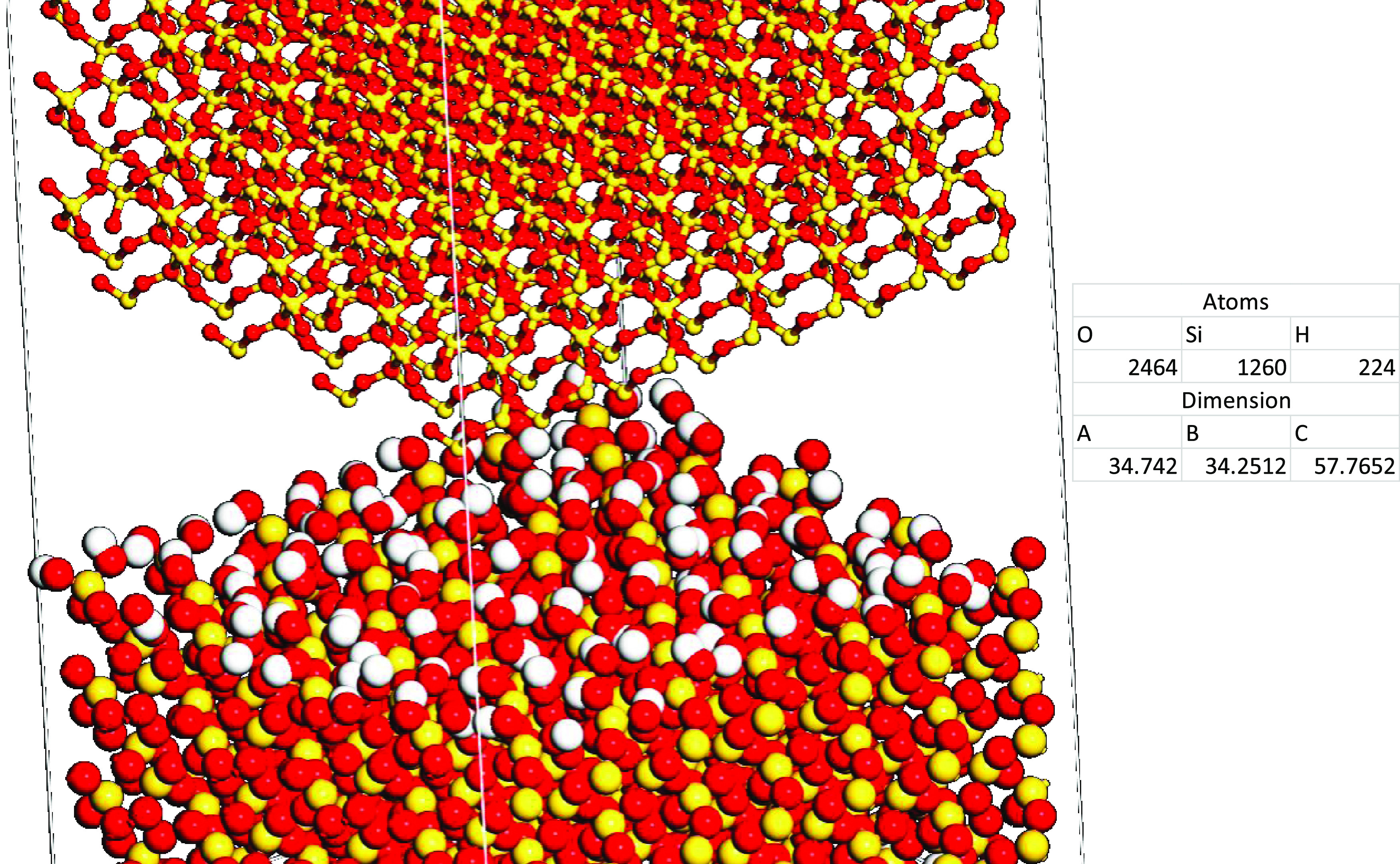
Illustration of the silica surface (down) and cement (up) adhesion model. Silicon (1260 atoms), yellow; oxygen (2464 atoms), red; and hydrogen (224 atoms), white.

Most cementing materials in reservoir rocks have been reported in the literature for both sandstones and limestones. They include carbonates (calcite, dolomite, and siderite), silicates (quartz and zeolite), sulfates (anhydrites and gypsum), and chlorides (halite), among others.^36^ In this study, only quartz was used as the cementing material for all surfaces. Hence, an α-quartz crystal was initially imported from the pre-existing Material Studio database and then converted to orthogonal geometry by cleaving in the direction of (0 0 1), since it has been reported to yield better results in model construction and conduction of dynamic simulations.^5^ Then, the dimensions of the cement were varied in accordance with the dimensions of each silica surface studied. Finally, the cement was placed over the surfaces with addition of vacuum between the silica surfaces and cement. Figure 2 illustrates the Q^2^ silica surface case with the cement placed over it.

Geometry optimization was then carried out, followed by an NVT simulation for 500 ps in all cases. The energy of the system was calculated by averaging points after reaching equilibrium.

To calculate the interaction energy between the surface and the cement, eq 3 was used.
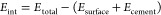
3where *E*_int_ is the interaction energy between the silica surface and cement, *E*_total_ is the total potential energy for the system, *E*_surface_ is the potential energy of the silica surface, and *E*_cement_ is the potential energy of the cement. The method of calculating the interaction energy (also called binding energy or adsorption energy) for a surface and a binder has been agreed upon and reported in the literature with successfully calculated values for adhesion of polymers, polymer over metals, and asphalt–silica systems.^16,37^

The interaction energy can be converted into adhesion pressure, a more related index in petroleum engineering, by simply dividing the interaction energy by the volume *V* of the cement and surface and using the appropriate conversion factor (see eq 4). Please also note that the work of adhesion, a typical property reported in the literature,^5^ is obtained in a similar manner, but by dividing the *E*_int_ by the surface area. This work focuses on the adhesion pressure since the comparison with experimental results is straightforward.
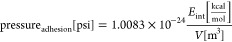
4where pressure_adhesion_ is the adhesion pressure at which the interaction between cement and surface fails and mechanical failure occurs.

#### 2.3.1 Water Effect Model

**Figure 3:**
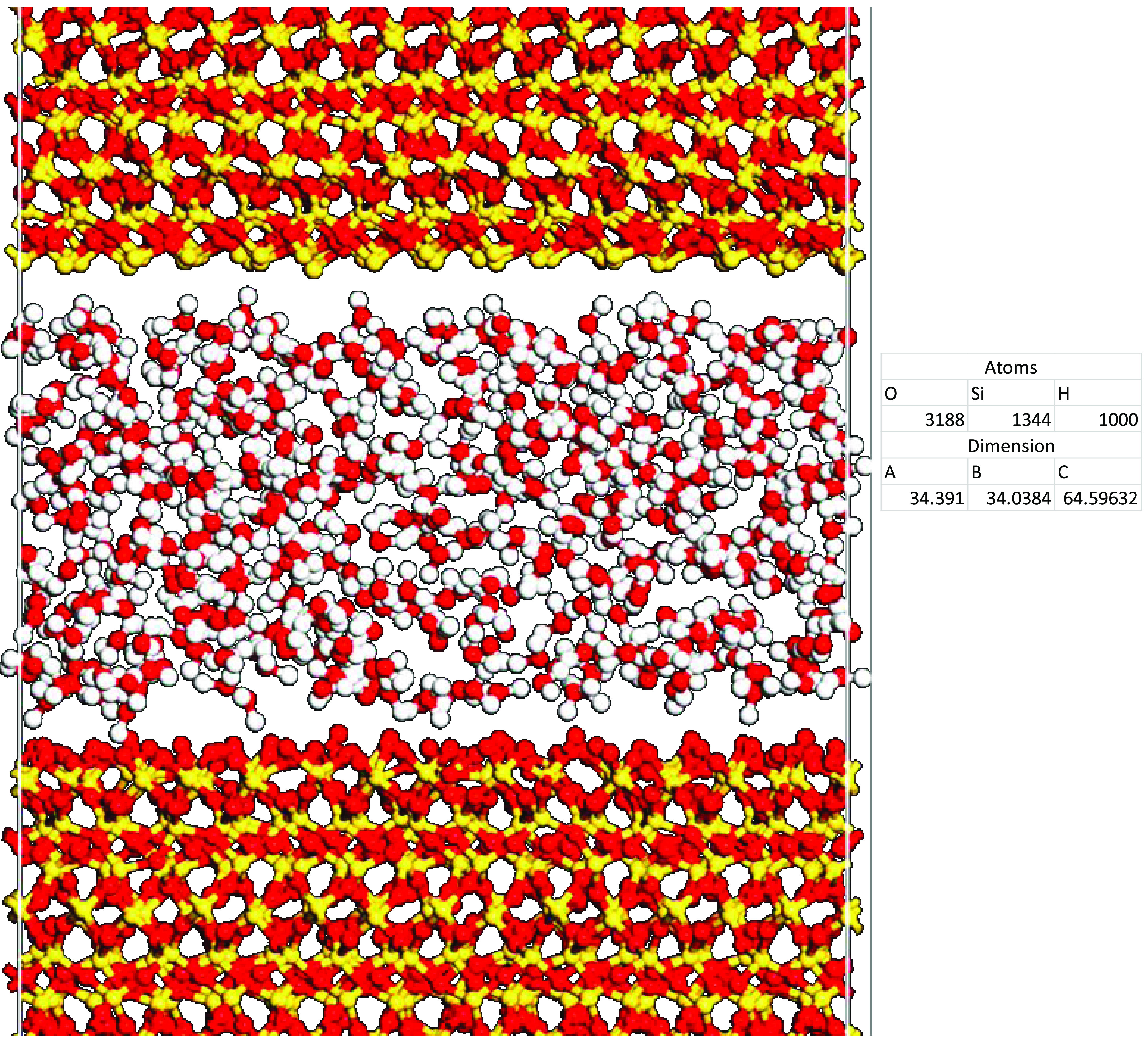
Water molecules between dry cement and silica surface. Silicon (1344 atoms), yellow; oxygen (3188 atoms), red; and hydrogen (1000 atoms), white.

The effect of water on the adhesion between the silica surfaces and cement was also investigated in this study. The base case silica surface was used in this section. Then, a vacuum of 30 Å was created between the surface and cement to insert different numbers of water molecules (i.e., 50, 100, 200, 300, and 500 molecules) in the interlayer space, as shown in Figure 3.

The geometry was then optimized and further equilibrated as previously mentioned for “dry” systems in the previous section. The influence of water is calculated based on the energy ratio. This term is widely reported in the literature to estimate the effect of moisture on the adhesion between a surface and a binding material. This is hypothesized as a direct proportionality between the sensitivity of moisture and dry adhesion bond energy and an inverse proportionality with debonding work.^38^ High values of energy ratio (more than unity) indicate the reduced tendency to damage the bonding at the interface. Lower values of energy ratio, on the other hand, indicate a damaging effect of water on the bonding between a surface and a binding material. The energy ratio is calculated as shown in eq 5(16)
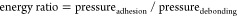
5where pressure_adhesion_ is the adhesion pressure between cement and surface on a “dry” system, and pressure_debonding_ is the pressure of debonding when including water in the systems, as H_2_O dissipates the energy at the surface–cement interface. Pressure_debonding_ is calculated using eq 6 after converting the debonding energy to debonding pressure using the previous steps.
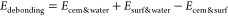
6where *E*_debonding_ is the debonding energy when water displaces cement from the surface–cement interface, *E*_cem&water_ is the interaction energy between the cement and the water, *E*_surf&water_ is the interaction energy between the surface and water, and *E*_cem&surf_ is the interaction energy between the cement and the surface of the same system.

## 3 Results and Discussion

In this section, the results obtained from the simulations are discussed in terms of the energy interactions between the silica surfaces for various silanol and ionized groups and quartz cement. Also, the cohesion of the cement and the surfaces is compared with the adhesion pressures.

**Figure 4:**
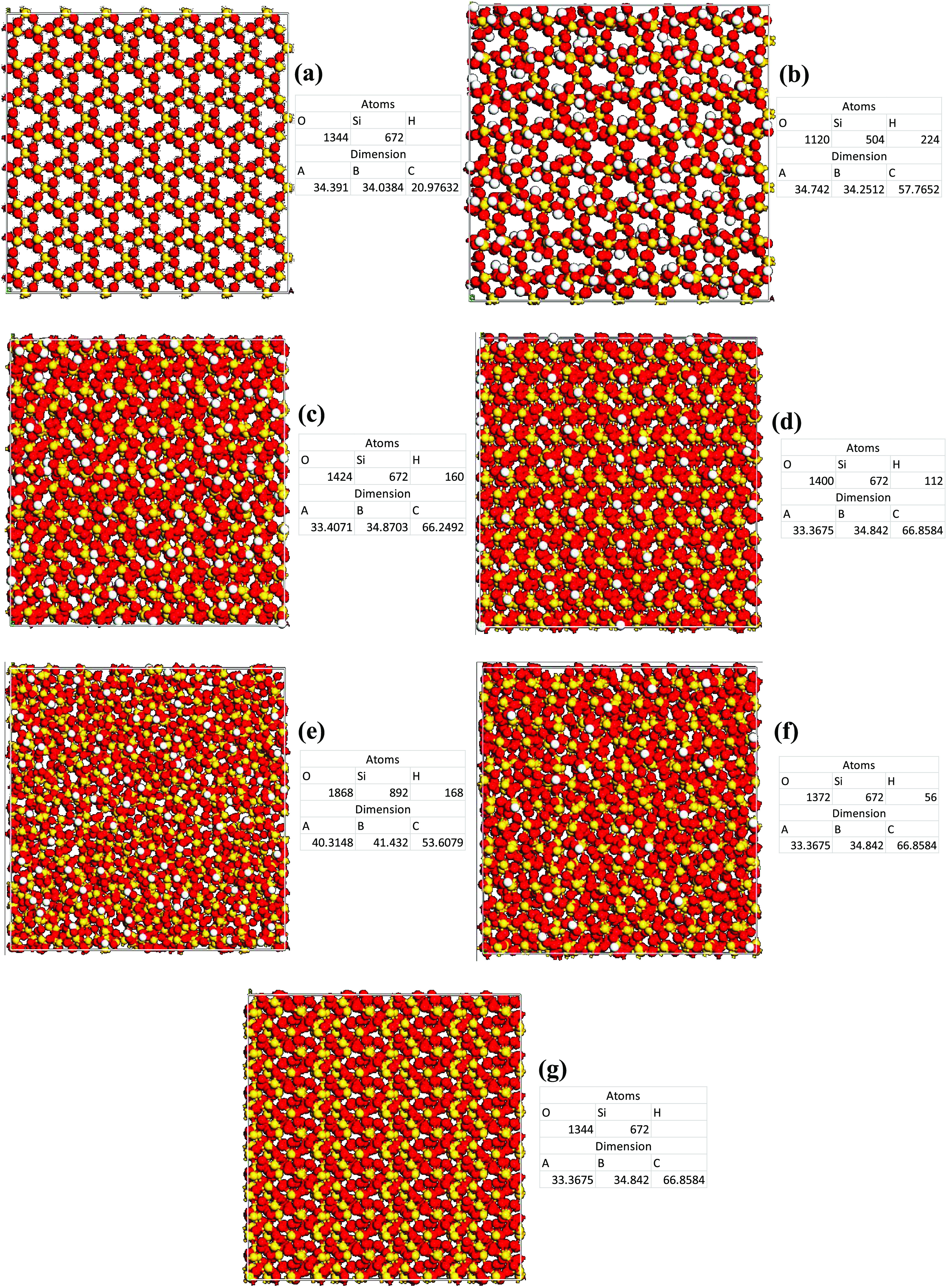
Silica surface configurations based on the silanol groups. (a) Base case (672 silicon atoms and 1344 oxygen atoms), (b) Q^2^ (504 silicon atoms, 1120 oxygen atoms, and 224 hydrogen atoms), (c) Q^2^-Q^3^ (672 silicon atoms, 1424 oxygen atoms, and 160 hydrogen atoms), (d) Q^3^ (672 silicon atoms, 1400 oxygen atoms, and 112 hydrogen atoms), (e) Q^3^ Amor (892 silicon atoms, 1868 oxygen atoms, and 168 hydrogen atoms), (f) Q^3^-Q^4^ (672 silicon atoms, 1372 oxygen atoms, and 56 hydrogen atoms), and (g) Q^4^ (672 silicon atoms and 1344 oxygen atoms).

Figure 4 shows seven different silica surface configurations based on the silanol groups used in this study. The models created are as follows: (1) base case representing a quartz cell with a rectangular shape with the alignment of the plane (0 0 1) with the z direction, (2) Q^2^ surface at silanol 9.4 per nm^2^, (3) Q^2^-Q^3^ surface at silanol 6.9 per nm^2^, (4) Q^3^ surface at silanol 4.7 per nm^2^, (5) Q^3^ amorphous surface at silanol 4.7 per nm^2^, (6) Q^2^-Q^3^ surface at silanol 2.4 per nm^2^, and (7) Q^2^ surface at silanol 0 per nm^2^.

### 3.1 Cohesion Pressure

Surface free energy is reported to be the amount of work needed to form a new unit area of an object. On an atomistic scale, the molecules of an object are encompassed from all directions with other molecules. In this manner, the surface free energy is equivalent to the work required to separate the molecules from the system by creating two surfaces for the extracted molecules and the bulk. Thus, the disturbance of the intermolecular interaction occurring with surface creation can be calculated using surface free energy. The literature reported some studies that use surface free energy and which gave good results to choose the best surface for cement combinations such as combining silica/calcite surfaces with an enhanced form of asphalt to improve the strength and the stability of the system.^39^ This type of result is referred to as the asphalt material cohesion bonding energy.

Surface free energy is used in this work to determine the cohesive pressure of the cement and the surface. The resulting surface free energy and cohesive pressure are shown in Table 3. It was found that the resulting cohesion pressures of the cement and the silanol surfaces fall in the range of a mechanical failure of sandstones indicating a possible representation of mechanical failure in sandstones.^40−42^

**Table 3:** Cohesion Pressure of the Cement and the Silica Surfaces at Different Silanol Configurations

cases	surface free energy (kcal/mol)	cohesion pressure (psi)
pure quartz	287.18	10048.4
Q^2^	160.66	6291.2
Q^2^-Q^3^	194.12	5737.7
Q^3^	61.40	1876.8
Q^3^ Amor	41.69	895.0
Q^3^-Q^4^	61.02	1847.4
Q^4^	193.26	6701.9

### 3.2 Adhesion Pressure

Although the reservoir rocks are visualized as connected grains of sands with cement such as quartz, the literature refers to the strength of the rocks as the cohesion of the rock.^43,44^ This means that the rock is considered as one solid porous object at which failure occurs when the cohesive strength is achieved (i.e., work to form a new unit area of an object is achieved). However, it is agreed that the grains in the rock are connected by a cement material. This kind of connection or bonding that holds the sand grains together cannot be represented as the cohesion strength of the rock.

**Figure 5:**
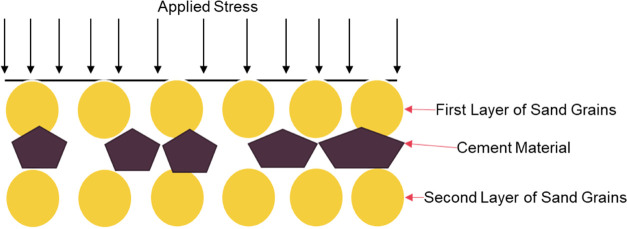
Nonuniform stress distribution on sand grains.

Currently, the rock is thought of as a cohesive body, meaning that it has one cohesive force that is holding it. Thus, based on this theory, if stress is applied to the rock, and based on the applied stresses and principal stresses, the failure of the rock should be uniform. However, this is not true; if we look inside a rock, we see that it consists of sand particles connected with a cement material. This can be supported by Potyondy et al. as they state that the cement-filled contacts experience compressive loads, and the force chains are highly nonuniform, with a few high-load chains and many low-load chains.^45^ The chain loads may be much higher than the applied loads, such that a few grains are highly loaded while others are less loaded or carry no load. This means that because of the cement existing between the sand grains, the load is nonuniformly transferred to other bonded grains as shown in Figure 5. Therefore, this is why we cannot say that the connection between the grains and cement is cohesion and can instead be thought of as adhesion; hence, we can observe that the failure creation in a rock is nonuniform.

**Figure 6:**
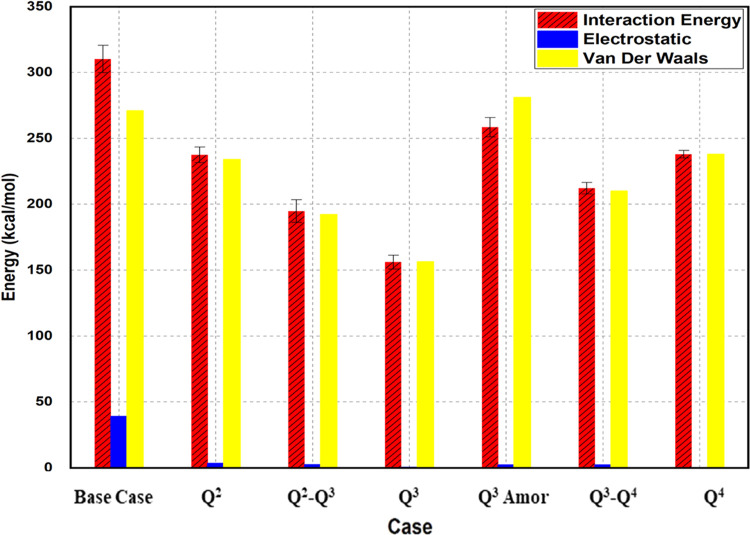
Nonbond energy contribution to the total interaction energy.

The adhesion pressure between the quartz cement and silica surfaces for different silanol configurations was investigated. The work of adhesion is reported in the literature as the work needed to separate two attracted bodies at the interface such as aggregates and asphalt.^16,37^ As previously mentioned, the work of adhesion in this study is converted into pressure (adhesion pressure), which can be further related to the resistance of the cement and the surface to the failure of the rock system (i.e., the fracturing of the rock). The variation in *E*_int_ between cement and different surfaces is mostly affected by the various silanol configurations. These values, which are the result of summing the electrostatic components and van der Waals interactions (as shown in Figure 6), generally show that the adhesion between cement and surfaces is resulted by the nonbonding interactions, noting that the covalent bonding is negligible. Figure 6 is obtained by plotting the nonbonding total energy, van der Waals, and electrostatic energies in kilo calories per mole for each of the silica surface configurations. The energy constituted by van der Waals interactions represented the highest contribution to the overall nonbonding energy. This indicates that the interaction between the cement and surface can be somewhat explained by physisorption rather than chemisorption. This can be visualized as cement adsorption onto the silica surface resulting in a somewhat condensate reaction between the cement and silica surface, which agrees with the fact that quartz cement is formed by precipitation and crystal overgrowth on the porous media.

Adding an adhesion bond between the quartz cement and silica grains in the rock system could reflect the actual behavior of sandstone rock. The failure of bonded grain assembly could also provide a representative system for the damage to the overall rock system. This gives a variation in the bonding strength depending on the strength of the adhesive bond between the cement and silica particles at different locations in the system.

Table 4 shows the results obtained from the calculation of the interaction energies and the corresponding adhesion pressure. It can be observed that all of the tested values of adhesion pressure fall in the range between 3000 and 6000 psi. This range agrees with most of the experimental failure tests on a large number of sandstones,^40−42^ corroborating the applicability of MD simulations as a useful and predicting tool for studying the failure of sandstone rocks.

**Table 4:** Interaction Energy and Corresponding Adhesion Pressure for Different Studied Silanol Surface Configurations

cases	interaction energy (kcal/mol)	adhesion pressure (psi)	description
base case	–310.19	6110.2	none silanol
Q^2^	–237.55	5076.0	9.4 silanol groups per nm^2^
Q^2^-Q^3^	–194.80	4162.5	6.9 silanol groups per nm^2^
Q^3^	–156.07	3030.9	4.7 silanol groups per nm^2^
Q^3^ Amor	–258.47	3493.7	4.7 silanol groups per nm^2^
Q^3^-Q^4^	–212.20	4121.0	2.4 silanol groups per nm^2^
Q^4^	–237.93	4837.5	0 silanol groups per nm^2^

#### 3.2.1 Comparison between Adhesion and Cohesion Pressure

**Figure 7:**
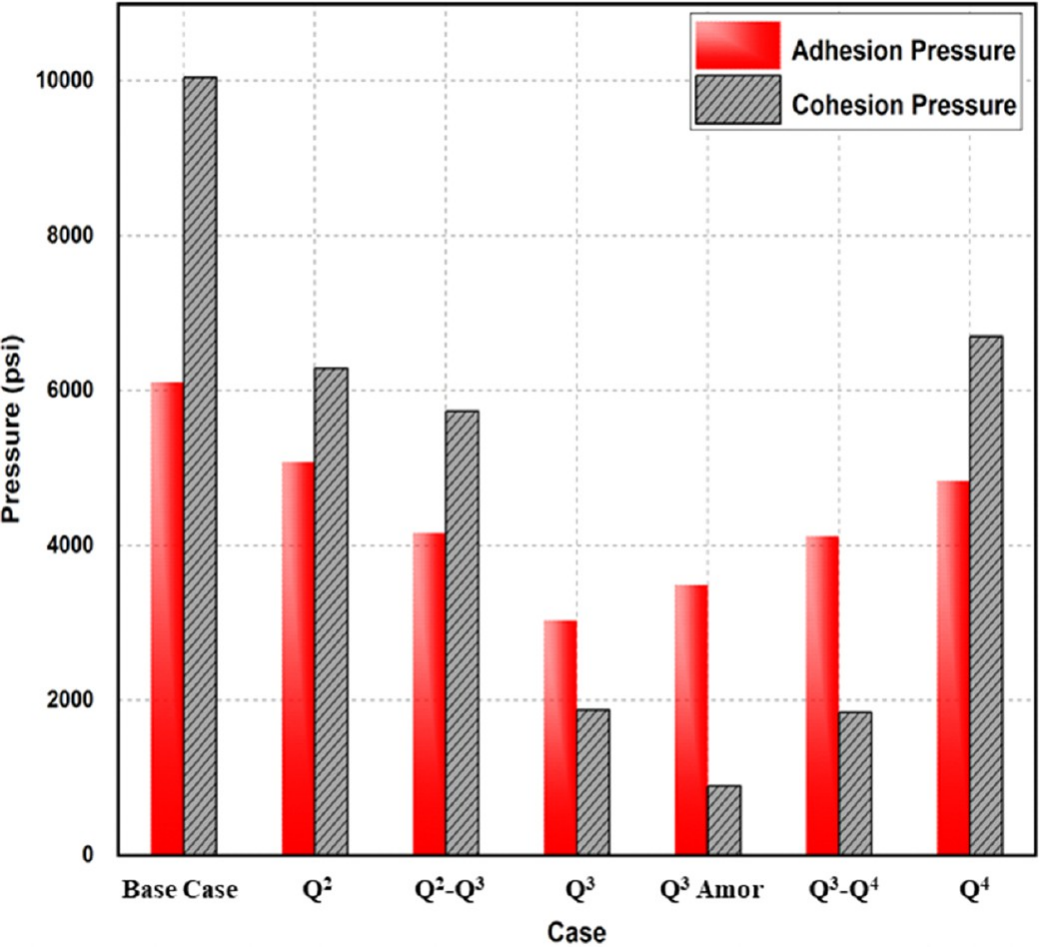
Comparison between adhesion and cohesion pressures for cement and silica surfaces at different silanol configurations.

To investigate whether the mechanical failure of a rock is caused by cohesion or adhesion, a comparison between the two pressures is provided as shown in Figure 7. Figure 7 is obtained by plotting the adhesion and cohesion pressures for each of the silica surface configurations. It is observed that the highest cohesion and adhesion values were obtained for the pure quartz case (10048.8 and 6110.2 psi for cohesion and adhesion pressure, respectively). The lowest values were obtained for the Q^3^ and Q^3^ amorphous cases. The strength of both cohesion and adhesion depended on the number of silanol groups and the configuration of the surface at the same time. Interestingly, the pure quartz, Q^2^, Q^2^-Q^3^, and Q^4^ cases have higher values of cohesion than those of adhesion. This could mean that mechanical failure occurs when the adhesion pressure is achieved. In this case, the reason for failure is the failure of the cement to hold two or more sand grains. On the other hand, Q^3^, Q^3^ amorphous, and Q^3^-Q^4^ resulted in lower cohesive pressure than the adhesion pressure. This might mean that for these three cases, mechanical failure occurs when the cohesion pressure is achieved. The cement or surface itself can fracture due to the lower surface free energy required for the sand grain or cement to fracture or break. This can explain the fact that most of the Q^3^ substrates form silica glasses and porous silica, which have lower cohesive strength compared to the Q^2^ and Q^4^ substrates forming quartz and silica surfaces.

These results indicate that mechanical failure is not always a cohesion failure but might also be an adhesion failure. This can give a new trend to investigate the effect of both adhesion and cohesion pressures on more detailed representative models for sandstones as well as other rock types such as limestones and shales, which will improve our understanding of mechanical failure and consolidation problems.

#### 3.2.2 Adhesion Pressure for Different Ionized Surfaces

The effect of ionization of a silica surface on the adhesive forces/pressures is also investigated in this study. This is due to the fact that a silica surface is typically in a protonation/deprotonation state. Protonation and deprotonation equilibria are common mechanisms occurring on Si–OH surfaces. At a point of zero charge (pH of 2–4), terminated surfaces of neutral silanol are achieved with a partial formation of metal siloxides. This partial formation occurs depending on various parameters such as the types of cations, ionic strength, and pH value. Siloxide is a chemical compound that is expressed by the term R_3_SiOM, where M refers to the metal cation. M usually ranges from 0 to 1 per silanol surface in nm^2^. This gives a value for the ionization of a Q^3^ surface of approximately 0–20% which corresponds for almost any silica surface to a value lower than 2.0 nm^2^ of the siloxide.

It is observed that ionization decreases with increasing the Q*^x^* configuration of the surface (decreasing from Q^2^ to Q^4^) corresponding to lower pH values and lower ionic strength. The literature reports that the most significant parameters for an ionized surface area depend mainly on area density Q^2^-Q^4^ and silanol group types discussed in the previous section, type of cations, ionic strength, and pH value.^34,46^

The two most significant parameters for building a silica surface are the substrate type, which represents the silanol areal density (which was investigated earlier), and the degree of ionization of the surface. Table 5 shows various models created to account for various ionization states of the silica surface with the corresponding interaction energy and adhesive force/pressure.

**Table 5:** Interaction Energy and Corresponding Adhesion Pressure For Different Ionized Silanol Surface Configurations

cases	interaction energy (kcal/mol)	adhesion pressure (psi)
Q^2^-1	–252.50	5395.4
Q^2^-2	–261.80	5594.1
Q^2^-Q^3^-1	–220.11	4703.3
Q^2^-Q^3^-2	–250.09	5344.0
Q^3^-1	–268.85	5221.2
Q^3^-2	–262.78	5103.3
Q^3^-3	–299.36	5813.6
Q^3^-4	–350.45	6805.9
Q^3^-5	–429.03	8331.9
Q^3^-6	–495.05	9614.0
Q^3^ Amor-1	–267.09	3610.3
Q^3^ Amor-1	–309.14	4178.6
Q^3^-Q^4^-1	–216.48	4204.0
Q^3^-Q^4^-2	–238.54	4632.5

**Figure 8:**
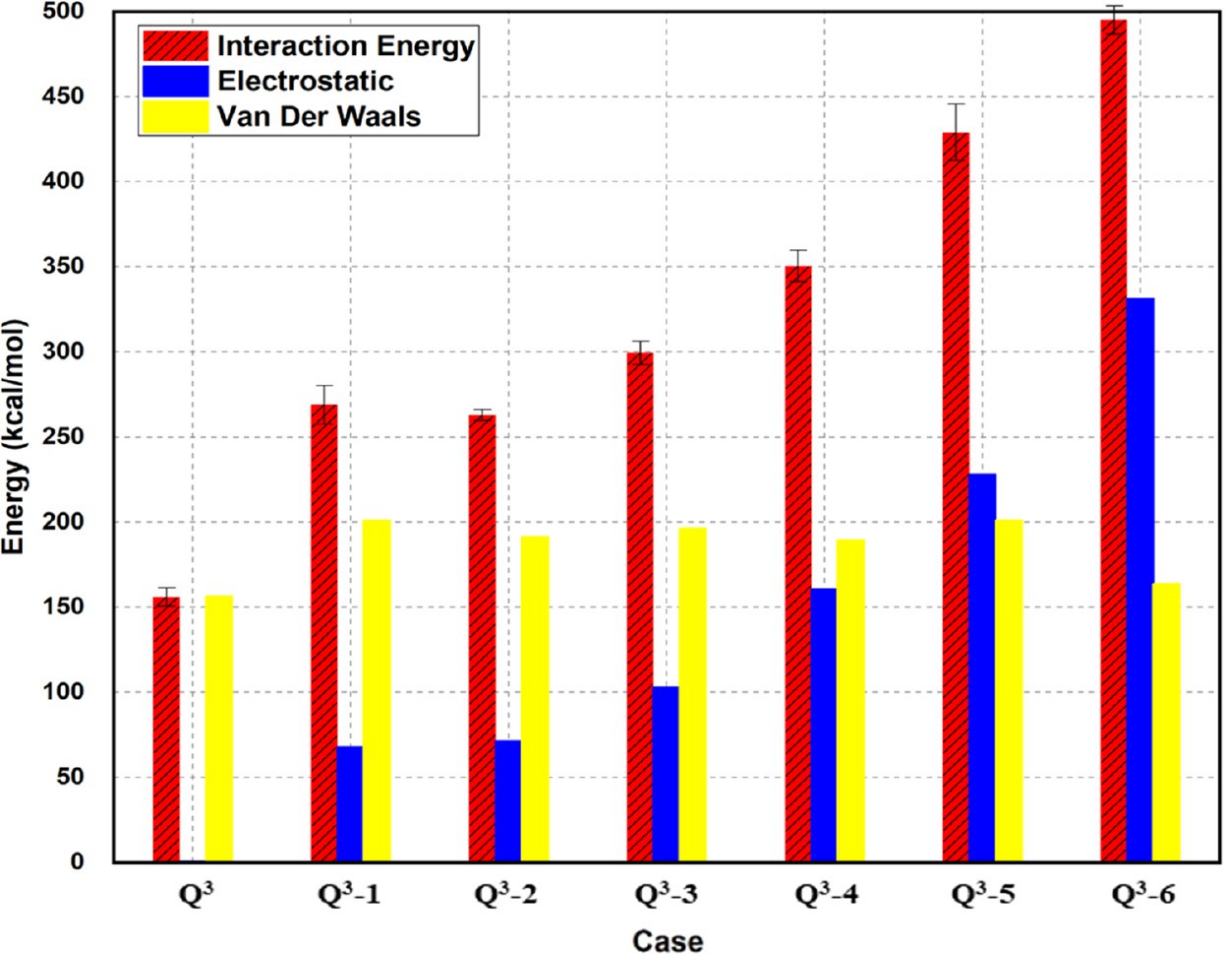
Nonbond energy contribution to the total interaction energy for the Q^3^ surfaces at different ionizations.

Overall, the interaction energy and hence the adhesive pressure increases with increasing the degree of ionization. The range of the results also falls in the range of mechanical failure of the sandstone rocks, which might indicate a possible prediction for the sandstone mechanical failure using MD simulations. To understand the increase of the interaction energy on ionizing the surface of the silica, we investigated the ionization effect on the interaction energy to restudy the nonbond forces. The values for the electrostatic and van der Waals components are shown in Figure 8. Figure 8 is obtained by plotting the nonbonding total energy, van der Waals, and electrostatic energies in kilo calories per mole for each of the Q^3^ silica surface configurations ionized at 0–50%. In this case, we noticed a considerable increase in the electrostatic forces as compared to the previous case in which the silica surface was not ionized.

**Figure 9:**
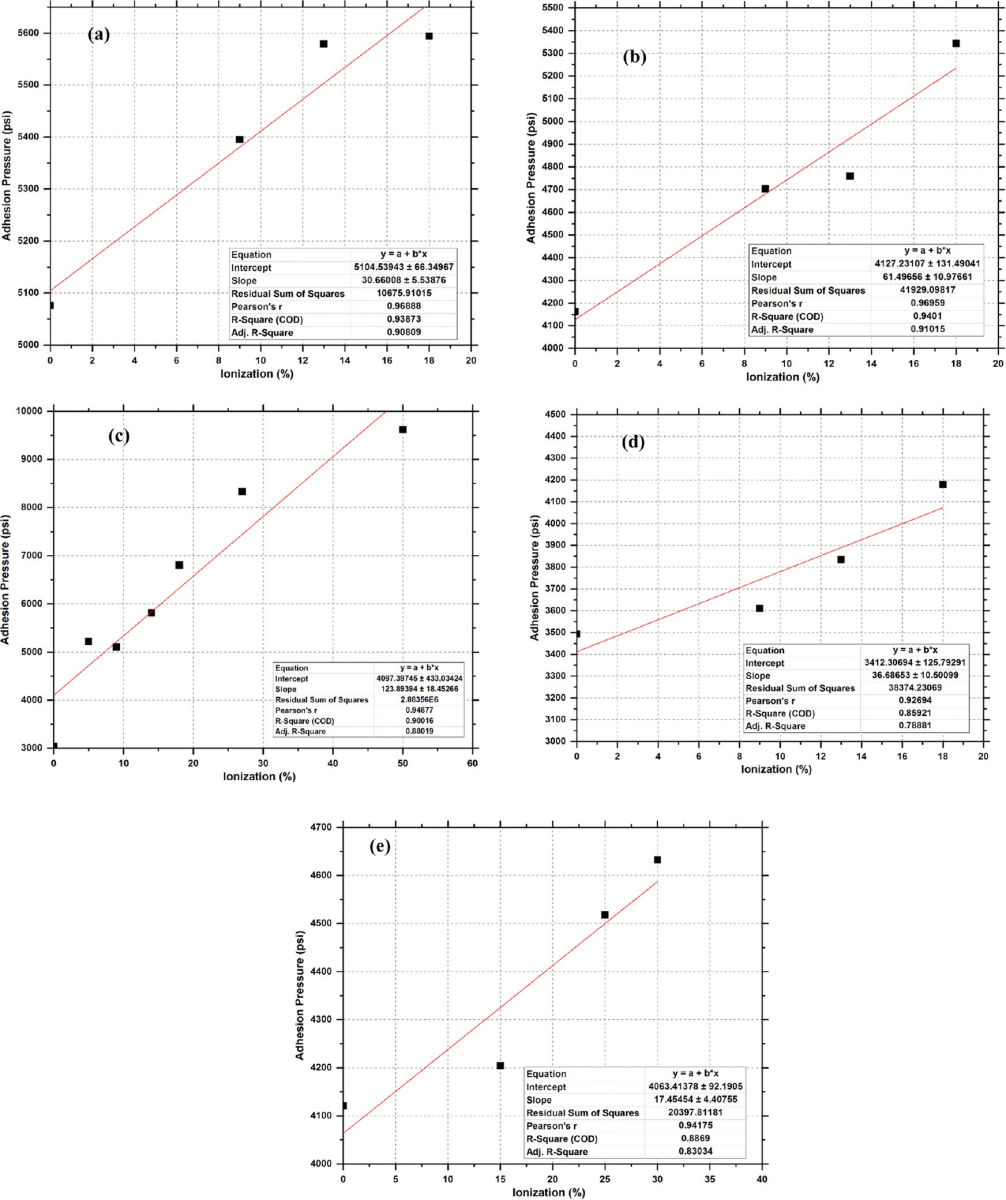
Effect of the degree of ionization on the adhesion pressure for (a) Q^2^, (b) Q^2^-Q^3^, (c) Q^3^, (d) Q^3^ Amor, and (e) Q^3^-Q^4^.

Figure 9 shows plots of the adhesion pressure versus the ionization degree generated for all of the ionized silica surface configurations in the range between 0 and 50%. Figure 9 graphically illustrates the effect of the degree of ionization on the adhesive forces at a specific hydroxylated degree of the surfaces. In all cases, at a fixed silanol value, the data correlated well between the degree of ionization and adhesive pressure. The overall effect of ionization on the adhesive pressure is the increase of the pressure with increasing ionization. This means that the interaction force between the cement and the silica surface can be increased by ionizing the silica surface. This might be a good indication of the possibility of increasing the consolidation of a sandstone rock for poorly consolidated reservoirs by means of ionizing the rock. This can mitigate/prevent excessive sand production from poorly consolidated (cemented) reservoir formations.

#### 3.2.3 Water Effect on Adhesion between Silica Surface and Cement

Silica surfaces have been widely investigated and reported in the literature. However, the impact of chemical topology and surface hydrophilicity on the interfacial water molecular properties is complex and not fully comprehended.^47^ The detrimental effect of water on the bonding between the cementitious material (e.g., quartz) and the silica surface is also investigated in this study. The amount of energy resulting in the disassociation or debonding of cement by water from the surface–cement interface is expressed here in terms of the debonding pressure as illustrated by eq 6. The debonding energies calculated in this study resulted in negative values for all of the energies and the corresponding debonding pressures, which indicate a thermodynamic potential of water disrupting the cement–surface interfaces.

**Figure 10:**
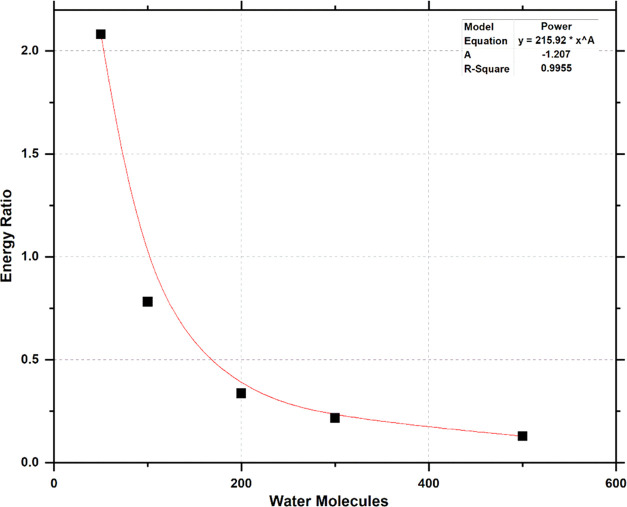
Effect of water molecules on the energy ratio.

The energy ratio in this work is calculated by dividing adhesion pressure in dry conditions by the adhesion pressure in wet conditions using eq 5. It is noteworthy to mention that it was assumed that introducing water to the model did not change the interaction area of the cement–surface interface and the water contacting fully with the cement and the surface at the interface. Figure 10 shows the plot of the number of water molecules ranging from 50 to 500 versus the calculated energy ratios. It shows the relationship between the number of molecules of water added to a silica surface–cement interface and the energy ratio. At a low number of water molecules (e.g., 50 molecules), the energy ratio is *ca.* 2. This indicates that the effect of water at the interface is small, which might indicate that there are still some higher interactions between the surface and cement. Increasing the number of water molecules to 100 molecules resulted in a steep decline in the energy ratio with a value of 0.781. At this number of water molecules at the interface between the cement and the dry surface, it is expected that the adhesive pressure between the silica surface and the cement is significantly reduced. Increasing the number of water molecules to more than 200 resulted in a less steep decrease in the energy ratio to a value below 0.336, which approaches an asymptotic behavior for the number of molecules above 500. This behavior is an indication of the formation of a disrupting thermodynamic potential of the silica surface–cement interfaces by the water. This means that because of the attractive nature of silica/quartz to water, a separation or a failure for the silica surface–cement interface could be initiated or achieved without the need to apply an external force.

Reservoir rocks are always exposed to water, which shows that the water effect on the rock surfaces always exists. This shows the importance of the adhesive pressure effect in the mechanical failure of rocks. Since the cores that are used in the experimental work lose their original water content after extraction from the reservoir, the experimental study is not able to represent the actual representation of the core in its original state. On the other hand, the original state of a core can be easily represented by the MD simulation with the help of various testing tools such as well logs and core analysis.

### 3.3 Correlating Adhesion Pressure with Area Ratio

**Figure 11:**
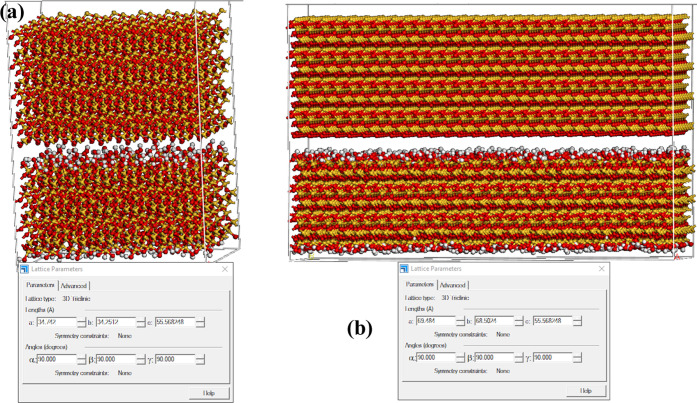
Silica surface and cement of (a) the original area and (b) the double size of the area.

The main problem faced by researchers is how to relate the smaller-scale properties to larger-scale properties or vis-versa. Calibrations are used to relate bulk properties such as Young’s modulus and Poisson’s ratio to calibrate microscale properties such as stiffness, which reduces the accuracy of the results. In this study, we tried to relate the adhesion pressure to a property that could be used on the nanoscale and larger scales. The first thing we observed is the similarity in adhesion pressure of a system consisting of a silica surface and a cement material with double areal size of the same system as shown in Figure 11.

**Figure 12:**
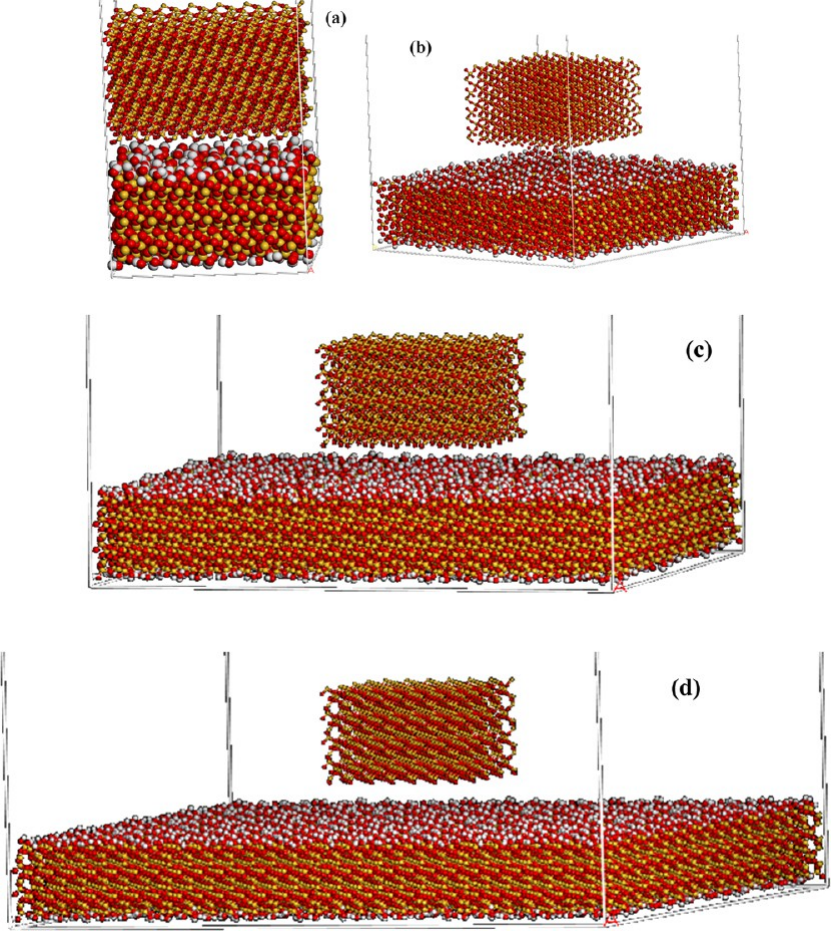
Quartz cement with silica surface of (a) similar area size, (b) double the size of the surface area, (c) a three times larger area, and (d) a four times larger area.

Figure 11 shows that the adhesion pressure is directly affected by the area of the cement and silica surface. To further investigate the effect of the area on the adhesion pressure, we created models with various silica surface sizes (Q^2^) and a fixed area size of the cement model as shown in Figure 12.

**Figure 13:**
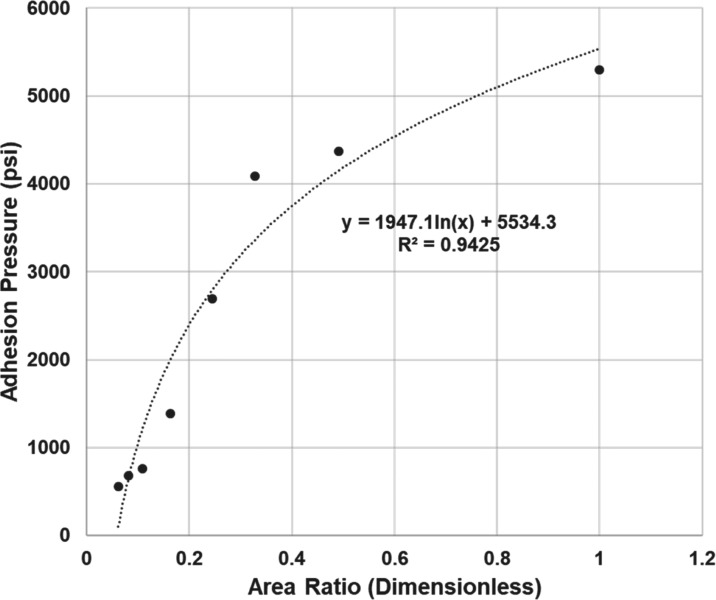
Correlation between the adhesion pressure and the dimensionless area ratio of cement quartz to the silica surface area.

The results showed a direct relationship between the adhesion pressure and the area ratio between the area of the cement and the area of the silica surfaces within the range of the sizes investigated. This typically falls in a range between consolidated to poorly consolidated sandstones as shown in Figure 13. The correlation allows for the distribution of adhesion pressure in systems at various scales by investigating the adhesion pressure at the nanoscale and distributing the resulting pressure based on the area ratio at the interface between the silica surface and the quartz cement at larger scales.

## 4 Conclusions

In this study, molecular dynamics simulations were used to investigate the adhesion, cohesion interactions, and water effect on adhesion between silica surfaces and quartz cement at a reservoir temperature of 260 °F and pressure of 4000 psi. The advantages of using MD are related to the lower cost and the possibility of predicting the mechanical failure of a reservoir rock at actual T,P conditions. In addition, studying the molecular interaction provides a fundamental understanding of the properties of a bulk system. The following observations were made.

- Resistance of the cement and the surface to the failure of the rock system is related to the adhesion pressure. The interaction between the silica surfaces and cement is a nonbond-component contribution represented mostly by van der Waals interactions. Adhesion pressure resulted for different silanol surfaces ranging between 3000 and 6000 psi, which agrees with a large number of experimental failure tests on most sandstones.
- Cohesion pressure was also calculated in this study resulting in an agreement with the range of mechanical failure of sandstones. Comparing the adhesion and cohesion pressures showed that adhesion can be the actual mechanism of the mechanical failure of a rock. Despite the reported literature considering cement as a significant component of the rock system, the rock system is visualized as a solid porous body with only cohesion forces holding it, which does not consider any adhesion forces between cement and grain surfaces.
- The adhesion pressure increased with increasing the degree of ionization. The range of the results was between 3500 and 9500 psi, which also falls in the range of the mechanical failure of sandstones.
- Energy ratio was used to investigate the effect of water on the adhesion forces between the silica surface and cement. It was found that water has a detrimental effect on adhesion. The additional reduction in adhesion by the water effect could also support the idea that the mechanical failure of the rock was caused by adhesion failure.
- Adhesion pressure can be correlated with the area ratio of the cement to the silica surface area and can represent larger-scale models.
